# The role of procalcitonin in neonatal intensive care unit patients with candidemia

**DOI:** 10.1007/s12223-012-0169-7

**Published:** 2012-06-12

**Authors:** Maria Teresa Montagna, Caterina Coretti, Antonella Rella, Giovanna Barbuti, Fabio Manca, Osvaldo Montagna, Nicola Laforgia, Giuseppina Caggiano

**Affiliations:** 1 Department of Biomedical Science and Human Oncology, University of Bari “Aldo Moro”, Piazza G. Cesare 11, 70124 Bari, Italy; 2 Department of Historical and Geographic Science, Statistic Unit, University of Bari “Aldo Moro”, Via Quintino Sella, 268-70123 Bari, Italy; 3 Department of Gynecology, Obstetrics and Neonatology, Neonatology and Neonatology Intensive Care Section, University of Bari “Aldo Moro”, Piazza G. Cesare 11, 70124 Bari, Italy

## Abstract

Candidemia is a major infectious complication in neonatal patients. The isolation of yeasts from blood is still the “gold standard” for its diagnosis, but other laboratory markers (i.e., circulating antigens) have been studied with varying specificities and sensitivities. The aim of this study was to evaluate the role of procalcitonin for the diagnosis of candidemia in neonatal patients at high risk. To verify if the use of different commercial methods can highlight dissimilar results of sensitivity and/or specificity, the determination of procalcitonin serum levels was estimated by two systems. Overall, 90 patients from a Neonatal Intensive Care Units were enrolled, of whom six developed *Candida* bloodstream infection. Four of six infants with candidemia had slight increase of procalcitonin values (0.5–1 ng/mL). Only one baby showed very high levels but he had fungal and bacterial sepsis at the same time, while no elevation was observed in the sixth patient. No statistically significant difference was observed between two different methods at the time of monitoring (*p* > 0.643). Both methods showed a sensitivity of 83.3 % at diagnosis, while the specificity was 73.8 and 63.1 % by methods A and B, respectively. In the light of the low sensibility and specificity of this assay, we can assume that the determination of procalcitonin would not seem to play a significant role in the diagnosis of fungal infection in neonatal patients.

## Introduction

The neonates admitted in Neonatal Intensive Care Unit (NICU) are highly prone to develop invasive fungal infections (IFIs) as result of the mostly unavoidable presence of several risk factors. The isolation of yeast from blood remains the “gold standard” for the diagnosis of candidemia in these patients, but it is hampered by small amount of blood that can be drawn from neonates (Connell et al. 2007; Rabalais et al. 1996; Smith and Congdon 1985), by nonspecific clinical symptoms and by frequent unavailability of *Candida* isolates from blood systems (their sensitivity for fungi may be as low as 50 %; Benjamin et al. 2000, 2003). In addition, blood cultures take some days to grow these organisms.

To date, other laboratory markers (C-reactive protein, *Candida* mannan, 1→3-beta-d-glucan antigen, specific antibodies, or DNA fungal detection) are currently considered as a useful support to achieve an early diagnosis of deep mycoses. However, some of these still require standardization and further evaluation and are not affordable for all laboratories because of various economic and technical reasons (Di Stefano et al. 2004; Oliveri et al. 2008; Mularoni et al. 2010).

Procalcitonin (PCT) is the precursor of the hormone calcitonin, normally produced by C-cells of the thyroid gland in response to hormonal stimuli and virtually undetectable in healthy subjects (<0.5 ng/mL). In response to inflammatory stimuli, the PCT can be also produced by liver (Nijsten et al. 2000) and peripheral blood mononuclear cells (Oberhoffer et al. 1999), increasing up to 1,000 ng/mL in patients with systemic bacterial infections or septic shock (Assicot et al. 1993; Meisner et al. 1999). Furthermore, it might help to distinguish bacteremia from non-infectious inflammatory conditions resulting in an important impact on therapeutic decisions (Meisner 2002; Simon et al. 2004; van Rossum et al. 2004; Schneider and Lam 2007; Becker et al. 2008).

Regarding the role of PCT in IFIs, the literature data are still few and controversial (Charles et al. 2009; Christofilopoulou et al. 2002; Huber et al. 1997); mostly, late elevations of PCT have been observed in patients with disseminated aspergillosis (Beaune et al. 1998) and in some children with invasive candidiasis (Dornbusch et al. 2005). Recently, Martini et al. (2010), studying the role of PCT in surgical patients with candidemia, observed that low PCT levels (less than 2.0 ng/mL) were more likely related to candidemia than bacteremia.

The aim of this study was to evaluate whether PCT may be an additional marker for *Candida*-related sepsis in neonatal patients at high risk of candidemia. To verify if the use of different commercial methods can highlight dissimilar results of sensitivity and/or specificity, the determination of PCT serum levels was estimated by two systems.

## Material and methods

The eligible patients were preterm infants (gestational age, <35 weeks) weighing ≤1,500 g at birth or full-term infants, all showing at least one of following clinical signs: apnea caused by respiratory stress, hypothermia, and tachycardia. Infants delivered by mothers receiving antibiotics either before or during delivery, not requiring intensive care, with mucocutaneous candidiasis or with proven bacterial sepsis were excluded from our study. Each enrolled patient was subjected to serum PCT assay from the third day of life (T3) and repeated on the fifth (T5), seventh (T7), ninth (T9), and 11th day (T11). Serum samples were kept refrigerated at −70 °C until they were processed. At the same interval time (T3, T5, T7, T9, and T11), blood cultures were carried out and an electronic report form was completed including clinical and microbiological data.

The determination of PCT serum levels was estimated in duplicate in every assay and on the same day by two systems: VIDAS® BRAHMS PCT (method A, bioMérieux, France) and KRYPTOR® BRAHMS PCT (method B, DASIT, Germany).

Method A is an automated test using the enzyme-linked fluorescent assay technique. It combines a one-step immunoassay sandwich method with a final fluorescent detection requiring 200 μL of serum per sample. Its measurement range is 0.05–200 ng/mL.

Method B is a homogeneous assay (sandwich principle) using TRACE® technology (time-resolved amplified cryptate emission). The needed serum volume is limited to 50 μL. The measuring range is 0.02–50 ng/mL.

We considered PCT values <0.5 ng/mL to be negative, ranging from 0.5 to 1.0 ng/mL moderately elevated, values >1.0 ng/mL highly elevated. Blood cultures were performed using a lyses centrifugation system (Isolator® DuPont Co, Delaware), incubated at 36 °C (±1 °C) for 7 days. Identification of yeasts was carried out by VITEK2 System (bioMérieux, France).

The day of notification of presence of yeasts in the blood culture was defined “infection day”. The data statistical analysis was carried out using the software Statistical Package for Social Sciences. The model used to obtain the significance of the averages during the time and among the different specimens is the ANOVA model (the variance analysis) which allowed us to calculate the *F* test and the *p* value <0.05. We used the technique of multiple comparison with the Tukey HSD model to analyze the significance of differences among groups. The tests gave information on which pairs of averages were significantly different, after a significant difference between averages of the groups was verified. Data for both methods were analyzed for sensitivity and specificity, negative predictive value (NPV) and positive predictive value (PPV).

## Results

**Fig. 1:**
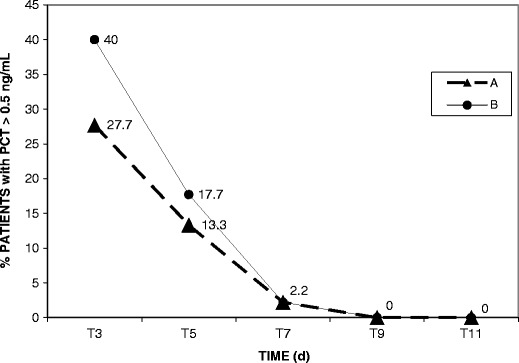
Percentage of NICU patients with PCT levels > 0.5 ng/mL in each time of monitoring, tested by Vidas® Brahms PCT, bioMérieux (Method A) and Kryptor® Brahms PCT, Dasit (Method B)

During the 18-months study, 90 infants were enrolled from NICU (Table 1), and a total of 447 sera were tested. Figure 1 shows the trend of 90 infants with PCT levels >0.5 ng/mL tested by A and B methods in each time. We observed that at time T3, the overall percentage of patients with PCT >0.5 ng/mL was 27.7 % by the method A and 40 % by the method B; these values progressively decreased until to 2.2 % at T7 and 0 % at T9 by both methods.

**Table 1:** Demographic and clinical characteristics of 90 enrolled NICU patients

Characteristics	Patients
	No. (%)
Sex	
Male	51 (56.6)
Female	39 (43.3)
Birth weight (g)	
≤1,000 (ELBW^a^)	14 (15.5)
1001–1500 (VLBW^b^)	76 (84.4)
Risk factors	
Gestational age <35 weeks	87 (96.6)
Central venous catheter	76 (84.4)
Respiratory distress syndrome	75 (83.3)
Parenteral nutrition	54 (60.0)
Mechanical ventilation	21 (23.3)
Others^c^	11 (12.2)

^a^Extremely low birth weight (body mass ≤1,000 g)

^b^Very low birth weight (body mass between 1,001 and 1,500 g)

^c^Nasogastric tube, hydrocephalus, and polycystic kidney

On the whole, six patients (6.6 %) developed a bloodstream infection (BSI): three caused by *Candida albicans* and three by *Candida parapsilosis* (Table 2). For each of these patients, the blood cultures resulted positive only one time during the period study. PCT values were slightly increased only in four patients (no. 1–4) either 2 days before or at diagnosis. Patient no. 5 showed very high values (17.1 and 7.02 ng/mL by methods A and B, respectively) but a severe bacterial BSI was confirmed at the same time, with an unfavorable outcome. For patient no. 6, the PCT values resulted always <0.5 ng/mL, with the exception of the assessment at the time T3 (0.62/0.53 ng/mL by A and B methods, respectively).

**Table 2:** PCT values in six babies recovered in NICU with *Candida* BSI tested by Vidas® Brahms PCT, bioMérieux (method A) and Kryptor® Brahms PCT, Dasit (method B)

Patients characteristics			PCT value (ng/mL) by A/B methods				
No./sex	Birth weight (g)	BSI agents	T3	T5	T7	T9	T11
1/M	1,230	*C. albicans*	0.48/0.33	0.69/0.53	0.58/0.52^a^	0.48/0.16	0.40/0.30
2/M	630	*C. parapsilosis*	0.98/0.60	1.27/0.80^a^	0.49/0.48	0.35/0.30	0.37/0.23
3/F	800	*C. albicans*	0.57/0.51	0.83/0.54^a^	0.44/0.42	0.35/0.35	0.20/0.30
4/M	1,150	*C. parapsilosis*	0.44/0.68	0.59/0.84	0.55/0.76^a^	0.17/0.36	0.06/0.14
5/M	1,310	*C. albicans*/*K. pneumoniae*	0.22/0.57	17.1/7.02^a^	Died		
6/F	1,130	*C. parapsilosis*	0.62/0.53	0.20/0.20	0.17/0.29	0.34/0.35^a^	0.22/0.18

^a^Infection day (day of notification of presence of yeasts in blood culture)

Considering the 84 uninfected newborns (Table 3), the serum PCT levels (the mean value ± SD) obtained with method A were <0.5 ng/mL (0.26 ± 0.09) in 62 patients and >0.5 ng/mL (0.56 ± 0.03) in 22 patients at time T3, <0.5 ng/mL (0.23 ± 0.09) in 77 infants and >0.5 ng/mL (0.53 ± 0.02) in seven infants at time T5. About the PCT levels obtained by the method B, the values resulted <0.5 ng/mL (0.22 ± 0.07) in 53 patients and >0.5 ng/mL (0.54 ± 0.03) in 31 patients at time T3, <0.5 ng/mL (0.20 ± 0.08) in 73 infants and >0.5 ng/ml (0.52 ± 0.01) in 11 infants at time T5.

**Table 3:** Number of NICU patients with PCT levels >0.5 and <0.5 ng/mL in each time of monitoring, tested by Vidas® Brahms PCT, bioMérieux (method A) and Kryptor® Brahms PCT, Dasit (method B)

PCT cut\-off value (ng/mL)	Patients (no.)				
Method A	^*^T3	T5	T7	T9	T11
>0.5	22	7	−	−	−
<0.5	62	77	84	84	84
Method B					
>0.5	31	11	−	−	−
<0.5	53	73	84	84	84

^a^T3, T5, T7, T9, and T11: times of PCT monitoring

The PCT values tested at time T7, T9, and T11 resulted always <0.5 ng/mL by the methods A and B in all the 84 uninfected patients. No statistically significant difference was observed between two methods at the time of monitoring (*p* > 0.643). Considering a threshold PCT value of 0.5 ng/mL and analyzing the patients showing slightly increased PCT, both methods showed a sensitivity of 83.3 % at diagnosis, while the specificity was 73.8 and 63.1 % by methods A and B, respectively. The NPV and PPV for the method A were 18.5 and 98.4 % respectively; for the method B were 13.9 and 98.1 %, respectively.

## Discussion

Although important progress is being made in the diagnosis of fungal diseases, *Candida* BSI still represents a clinical event of difficult resolution. In NICU patients, the early detection of sepsis is difficult because the first signs of this disease may be minimal or similar to those of non-infectious processes. Furthermore, the results of blood cultures are not quickly available and other laboratory tests lack high sensitivity and specificity.

Recently, some authors have considered the PCT value as a potential biological marker of fungal sepsis (Charles et al. 2009; Christofilopoulou et al. 2002; Martini et al. 2010). Unfortunately, its role in diagnosis of these diseases has not yet been elucidated, particularly regarding newborns: they may exhibit elevated PCT levels because of a physiological increase in the first days of life which revert to normal range 3–4 days after birth (Turner et al. 2006). This occurrence could be caused by birth trauma or host response to the initial establishment of the normal intestinal bacterial flora or by the adaptation to the extra-uterine environment. These phenomena stimulate an acute phase reaction with release of C-reactive protein, interleukin-6, and serum amyloidal A (Chiesa et al. 2001; Marchini et al. 2000). For this reason, we excluded patients younger than 72 h from our research.

Our findings showed a slight increase of PCT values both in some infants without fungal infections as in four out of six infants with BSI. In the light of these results, it is difficult to understand the role of PCT in the diagnosis of *Candida* BSI in NICU patients. In fact, its low sensibility and specificity leads us to consider that PCT determination does not play a significant role in the diagnosis of these infections respect to other diagnostic assays. In particular, in another study, 1→3-beta-d-glucan (BDG) and *Candida* mannan (CM) were tested in the same neonates with *Candida* BSI only at the time as the positive blood cultures: in all six patients, the BDG test resulted positive, while the CM antigen resulted positive only in three patients with *C. albicans* BSI (Montagna et al. 2011).

We are aware that this study presents the limitation of the low number of patients with candidemia, but it is well known that obtaining appropriate and seriated specimens in neonatal patients is hampered by the critical conditions of these patients and by small amount of blood that can be drawn.

Regarding the two methods used to evaluate PCT levels, no statistically significant differences was detected (*p* > 0.05); nevertheless, we want to underline some questions. If we consider PCT values, <0.5 ng/mL to be negative and ranging from 0.5 to 1.0 ng/mL to be moderately elevated and analyze the individual values of PCT detected in any patient with candidemia, some discrepancies can appear between the two methods. In fact, the two systems employed at the same time and on the same serum sample of two out of six infants with candidemia (patients no. 4 and 5) highlight negative or slightly positive values respectively, posing a problem for the evaluation of PCT and interpretation of data: no fungal sepsis could be suspected for the infants no. 4 and 5 at time T3 if we consider the PCT values obtained by method A (0.44 and 0.22 ng/mL, respectively), but a doubt appears in the same patients if we consider the PCT value obtained by method B (0.68 and 0.57 ng/mL, respectively). Therefore, in these two cases, the two methods do not lead to the same clinical interpretation. Besides, for infant no. 4, the PCT values obtained by both methods appear to differ also at time T5 (0.59 and 0.84 ng/mL, respectively) and T7 (0.55 and 0.76 ng/mL, respectively). Perhaps, it could be useful to recognize an appropriate cutoff concentration for the fungal infections both to exclude false interpretations from use of different commercial systems and to separate fungal infections from bacterial ones.

## Conclusions

Our data suggest that PCT assay do not play a significant role in neonates with candidemia. We can assume that its values from 0.5 to 1 ng/mL cannot be considered a reliable indicator of fungal sepsis, as well as low values of PCT cannot exclude this infection. We consider our results preliminary data carried out in a single neonatal intensive care unit planning to follow a line of a multicenter investigation.
